# Genome-Wide DNA Methylation Analysis Reveals a Potential Mechanism for the Pathogenesis and Development of Uterine Leiomyomas

**DOI:** 10.1371/journal.pone.0066632

**Published:** 2013-06-20

**Authors:** Ryo Maekawa, Shun Sato, Yoshiaki Yamagata, Hiromi Asada, Isao Tamura, Lifa Lee, Maki Okada, Hiroshi Tamura, Eiichi Takaki, Akira Nakai, Norihiro Sugino

**Affiliations:** 1 Department of Obstetrics and Gynecology, Yamaguchi University Graduate School of Medicine, Ube, Yamaguchi, Japan; 2 Department of Biochemistry and Molecular Biology, Yamaguchi University Graduate School of Medicine, Ube, Yamaguchi, Japan; Sapporo Medical University, Japan

## Abstract

**Background:**

The pathogenesis of uterine leiomyomas, the most common benign tumor in women, remains unclear. Since acquired factors such as obesity, hypertension and early menarche place women at greater risk for uterine leiomyomas, uterine leiomyomas may be associated with epigenetic abnormalities that are caused by unfavorable environmental exposures.

**Principal Findings:**

Profiles of genome-wide DNA methylation and mRNA expression were investigated in leiomyomas and in myometrium with and without leiomyomas. Profiles of DNA methylation and mRNA expression in the myometrium with and without leiomyomas were quite similar while those in leiomyomas were distinct. We identified 120 genes whose DNA methylation and mRNA expression patterns differed between leiomyomas and the adjacent myometrium. The biological relevance of the aberrantly methylated and expressed genes was cancer process, including IRS1 that is related to transformation, and collagen-related genes such as COL4A1, COL4A2 and COL6A3. We also detected 22 target genes of estrogen receptor (ER) alpha, including apoptosis-related genes, that have aberrant DNA methylation in the promoter, suggesting that the aberrant epigenetic regulation of ER alpha-target genes contributes to the aberrant response to estrogen.

**Conclusions:**

Aberrant DNA methylation and its related transcriptional aberration were associated with cancer processes, which may represent a critical initial mechanism that triggers transformation of a single tumor stem cell that will eventually develop into a monoclonal leiomyoma tumor. The aberrant epigenetic regulation of ER alpha-target genes also may contribute to the aberrant response to estrogen, which is involved in the development of uterine leiomyomas after menarche.

## Introduction

Uterine leiomyomas are the most common uterine tumors in reproductive-age women with a prevalence of about 25% [1]. Uterine leiomyomas frequently cause serious gynecological problems such as pelvic pain, menorrhagia, dysmenorrhea, infertility and recurrent pregnancy loss [1], [2]. In addition, uterine leiomyomas are the most common indication for hysterectomy. Risk factors for uterine leiomyomas include African descent, high body mass index, meat consumption, early menarche, hypertension and a history of pelvic inflammatory disease. On the other hand, factors that lower the risk include use of hormonal contraception, smoking, giving birth and consumption of green vegetables [3], [4], [5]. These findings suggest that both genetic and environmental factors are involved in the development of uterine leiomyomas. In addition, uterine leiomyomas often show multifocal tumorigenesis with various sizes from the corresponding myometrium. These findings suggest that smooth muscle cells of normal myometrium in the uterus with leiomyomas already acquire the potential in molecular levels to develop into leiomyomas in future.

DNA methylation is one of the well characterized epigenetic marks and plays a crucial role in the regulation of gene expression. DNA methylation is specific to each cell type and has been used to characterize abnormal cells [6], [7], [8]. Maintaining the specific DNA methylation profile of the cell is necessary for cellular integrity, and alterations in DNA methylation may have serious health consequences. Environmental factors can be shown to affect DNA methylation [9], [10]. We previously demonstrated that uterine leiomyomas have an aberrant DNA methylation profile using genome-wide DNA methylation analysis methods [11], [12]. Navarro et al. also analyzed the DNA methylation patterns in uterine leiomyomas using genome-wide analysis method in African American women [13]. They found several aberrantly methylated and concomitantly expressed genes in leiomyomas, and suggested that DNA methylation plays a key role in the pathogenesis of uterine leiomyomas by altering the normal myometrial mRNA expression profile. The study by Navarro et al. examined one or two CpG sites in the promoter region in each gene. Recently, a new type of genome-wide DNA methylation analysis has been developed, which covers about 16 CpG sites from the promoter to the gene body region. Therefore, the new analysis will provide more detailed information on genome-wide DNA methylation profiles in uterine leiomyomas.

Although uterine leiomyomas develop only after menarche and their growth depends on estrogen, the mechanism is not fully clarified. The biological effect of estrogen is mediated by estrogen receptor (ER), which is a transcription factor [1]. DNA methylation of the gene promoter region directly affects the response to transcription factors [14]. Thus, aberrant DNA methylation of the promoter regions of genes targeted by ER alpha directly may affect the response to estrogen. Recently, exposure to environmental estrogen during reproductive tract development has been shown to cause genes targeted by ER alpha to become hyper-responsive to estrogen in the adult myometrium [15]. These findings raise the possibility that aberrant DNA methylation of the promoter of ER alpha-target genes may cause the aberrant responses to estrogen exposure in uterine leiomyomas, which may be involved in the development of uterine leiomyomas after menarche.

We previously reported that aberrant DNA hypomethylation is enriched on the X chromosome in uterine leiomyomas compared with the adjacent normal myometrium [12]. Interestingly, the X chromosome was found to have more hypomethylated genes than other chromosomes [12]. It has been reported that breast cancers, ovarian cancers, and cervical cancers have aberrant DNA hypomethylation on the X chromosome such as loss of inactive X chromosome or aberrant replication of active X chromosome [16], [17]. Therefore, an analysis of the genotype of the X chromosome might provide clues to the cause of uterine leiomyomas.

The main objective of this study is to determine whether DNA methylation patterns are associated with the pathogenesis and development of uterine leiomyomas. To this end, we 1) compared the DNA methylation profiles and mRNA expression profiles among uterine leiomyomas and myometrium with or without leiomyomas using an advanced genome-wide DNA methylation analysis method, 2) attempted to identify a subset of genes whose differential DNA methylation in the promoter is correlated with differential mRNA expression, 3) identified ER alpha-target genes that have aberrant DNA methylation in the promoter, and 4) examined the genotype of the X chromosome and the chromosome distribution of aberrant DNA methylation in uterine leiomyomas.

## Materials and Methods

### Ethics Statement

This study was reviewed and approved by the Institutional Review Board of Yamaguchi University Graduate School of Medicine. Written informed consent was obtained from the participants before the collection of any samples, and the specimens were irreversibly de-identified. All experiments handling human tissues were performed in accordance with Tenets of the Declaration of Helsinki.

### Tissue Preparations

Paired specimens of leiomyoma and adjacent normal myometrium were obtained from 10 Japanese women who had a single leiomyoma nodule to limit biological heterogeneity (Table 1). The women underwent hysterectomy, and their ages were 44.1+/−4.5 years old (mean +/− SD). The size of leiomyoma ranged from 40 to 160 mm (mean +/− SD; 85.5+/−34.0 mm). All patients showed intramural leiomyoma. As control myometrium tissues, we obtained normal myometrium tissue from 3 women who underwent hysterectomy under the diagnosis of early stage of cervical cancer (Control 1, Control and Control 3 in Table 1). The ages of control women were 38.3+/−5.0 years old (mean +/− SD), and did not significantly differ from women with uterine leiomyomas. None of the women had received previous treatment with sex steroid hormones or gonadotropin releasing hormone analogs. Specimens were dissected immediately from the full thickness uterine wall without the endometrium and serosa after removal of the uterus, immersed in liquid nitrogen and stored at -80°C until DNA/RNA extraction. Leiomyomas (L1, L2 and L3) and adjacent myometrium (M1, M2 and M3) obtained from 3 cases (Case 1, Case 2 and Case 3 in Table 1) were examined by following genome-wide DNA methylation analysis and mRNA expression analysis. The myometrium from 3 control women were also examined (C1, C2, and C3). The other 7 paired samples of leiomyoma and adjacent myometrium were used in bisulfite restriction mapping and RT-PCR.

**Table 1:** Patients with uterine leiomyomas and characteristics of their leiomyomas.

	Age (y.o.)	Menstrual phase	Type	Diameter (mm)
Control 1	43	Follicular	–	
Control 2	33	Follicular	–	
Control 3	39	Luteal	–	
Case 1	49	Luteal	intramural	160
Case 2	49	Follicular	intramural	90
Case 3	40	Luteal	intramural	80
Case 4	43	Follicular	intramural	90
Case 5	39	Follicular	intramural	70
Case 6	40	Follicular	intramural	85
Case 7	38	Luteal	intramural	120
Case 8	48	Follicular	intramural	40
Case 9	47	Follicular	intramural	60
Case 10	48	Luteal	intramural	60

Controls 1–3 and Cases 1–3 were used for analysis with Illumina Infinium HumanMethylation450 BeadChip and Human Gene 1.0 ST array. Cases 1–10 were used for validation of the genes with differential DNA methylation and mRNA expression.

### Illumina Infinium HumanMethylation450 BeadChip Assay

Genomic DNA was isolated from 20 mg frozen tissues using the Qiagen Genomic DNA (Qiagen, Valencia, CA, USA). The DNA methylation analysis was performed using the Illumina infinium assay with the HumanMethylation450 BeadChip (Illumina, San Diego, CA, USA), which interrogates a total of 482,421 CpG sites spread across the distal promoter regions of transcription start sites to 3′-UTR of consensus coding sequences. Methylated and unmethylated signals were used to compute beta values, which are quantitative scores of the DNA methylation levels, ranging from “0,”, thus indicating completely unmethylated, to “1,” indicating completely methylated. The BeadChip was scanned on a BeadArray Reader (Illumina) according to the manufacturer’s instructions. CpG sites with “detection p values” >0.05 (computed from the background based on negative controls) and CpG sites on Y chromosome were eliminated from further analysis, leaving 482,005 CpGs valid for use with the nine samples tested. To perform hierarchical clustering analysis and principal component analysis, we extracted the CpG sites in which the difference of maximum beta value and minimum beta value in 9 tissues is greater than 0.3. To identify the CpGs that showed methylation changes between leiomyoma and adjacent myometrium, the difference of beta value of CpGs were calculated in each case. Then, CpGs expressing greater than 0.3 were extracted. The microarray data of DNA methylation is available at the Gene Expression Omnibus Web site (http://www.ncbi.nlm.nih.gov/geo/) under accession No. GSE45187.

### Transcriptome Analysis

Gene expression was analyzed using a GeneChip® Human Gene 1.0ST Array (Affymetrix, Santa Clara, CA, USA) containing 764,885 probes supporting 28,869 genes. Target cDNA was prepared from 200 ng of total RNA with the Ambion® WT Expression kit (Ambion, Austin, TX, USA) and the Affymetrix® GeneChip WT Terminal Labeling kit (Affymetrix). Hybridization to the microarrays, washing, staining and scanning were performed using the GeneChip® system (Affymetrix) composed of the Scanner 3000 7G Workstation Fluidics 450 and the Hybridization Oven 645. The scanned image data were processed using a Gene Expression Analysis with the Partek® Genomics Suite 6.5 software program (Partech, Munster, Germany). To perform hierarchical clustering analysis and principal component analysis, we extracted the gene in which the ratio of maximum value to minimum value in 9 tissues is greater than 1.5-fold. To identify the genes that show the difference in mRNA expression between leiomyoma and adjacent myometrium, a fold-change greater than 1.5-fold or lesser than 0.67-fold was recognized as a significant difference. The microarray data of mRNA expression is available at the Gene Expression Omnibus Web site (http://www.ncbi.nlm.nih.gov/geo/) under accession No. GSE45188.

### Bisulfite Restriction Mapping

Bisulfite reactions were performed as previously reported [18]. The bisulfite-converted DNA was amplified by PCR using the following sets of primers: IRS1 F: 5′-TGTTTTATTTTAAATTTTTAGTGGAGAGTAG-3′, R: 5′-AATATCCCCCACCCAAACTATC-3′; COL4A1 F: 5′-GGAGGGAGGTAGAGGATTTATGT-3′, R: 5′-AAAAACTCCTAAATAAATCTAAAAAAAACT-3′; GSTM5 F: 5′-GGGAGGGGGTTTATTGATTTTAGTT-3′, R: 5′-ACCAATCCTACAACTACTCCACACATC-3′. The thermocycling program was an initial cycle of 94°C for 10 min, then 43 cycles of 94°C for 30 sec, 59°C for 30 sec and 72°C for 1 min and finally 10 min of final extension at 72°C. For restriction mapping, 1/2 each of the PCR products was treated with or without restriction enzyme, and the resulting DNA fragments were assessed by agarose gel electrophoresis.

### Real-time Quantitative RT-PCR

Total RNAs were isolated from tissues using TRIzol reagent (Invitrogen, Carlsbad, CA, USA) and reverse-transcribed using a QuantiTect Reverse Transcription Kit (Qiagen) according to the manufacturer’s protocol. The synthesized DNA was subjected to PCR reactions using the following sets of primers: IRS1 F: 5′- CAAGACCATCAGCTTCGTGA -3′, R: 5′- AGAGTCATCCACCTGCATCC -3′; COL4A1 F: 5′- TGGTGACAAAGGACAAGCAG -3′, R: 5′- GGTTCACCCTTTGGACCTG -3′; GSTM5 F: 5′- AATGCCATCCTGCGCTAC -3′, R: 5′- TTCTCCAAAATGTCCACACG -3′. All PCRs were performed using SYBR Premix Ex Taq (TAKARA) and a LightCycler (Roche Applied Science, Basel, Switzerland). A primer set for glyceraldehyde-3-phosphate dehydrogenase (GAPDH) was used as an internal control. All samples were run in duplicate. Melting curves of the products were obtained after cycling by a stepwise increase of temperature from 55 to 95°C. At the end of 40 cycles, reaction products were separated electrophoretically on an agarose gel and stained with ethidium bromide for visual conformation of the PCR products.

### X chromosome Genotyping

The analysis was done using a panel of primers specific for the following X-linked short tandem repeats markers, obtained from Genome Database (http://www.gdb.org/) and National Center for Biotechnology Information (http://www.ncbi.nlm.nih.gov/genomes/static/euk_g.html): DXS7132, DXS6789, DXS8377 and DXS6807 spanning p and q arms. After PCR using one fluorescent-labeled primer, the products were separated electrophoretically on the Fluorescent Capillary System ABI PRISM 310 and analyzed with GeneScan software (Applied Biosystems). The allelic status in each case was examined by comparing the allelic statuses of leiomyoma and adjacent myometrium.

### Bioinformatics

MultiExperiment Viewer (MeV in the TM4 microarray Software Suite; http://www.tm4org/mevhtml) was used for principal component analysis (PCA) [19]. The analysis for ER alpha-target genes analysis was performed using the MAPPER database (http://mapper.chip.org/) [20]. Chromosome distribution analysis was performed using DAVID 6.7 (http://david.abcc.ncifcrf.gov/home.jsp) [21]. Integrative Genomics Viewer (http://www.broadinstitute.org/igv/) was used for chromosomal distribution map generation [22]. QUMA (http://quma.cdb.riken.jp/) was used to analyze the bisulfite sequencing data [23].

## Results

### Analysis of DNA Methylation Profiles of Leiomyomas and Myometrium with and without Leiomyomas

**Figure 1:**
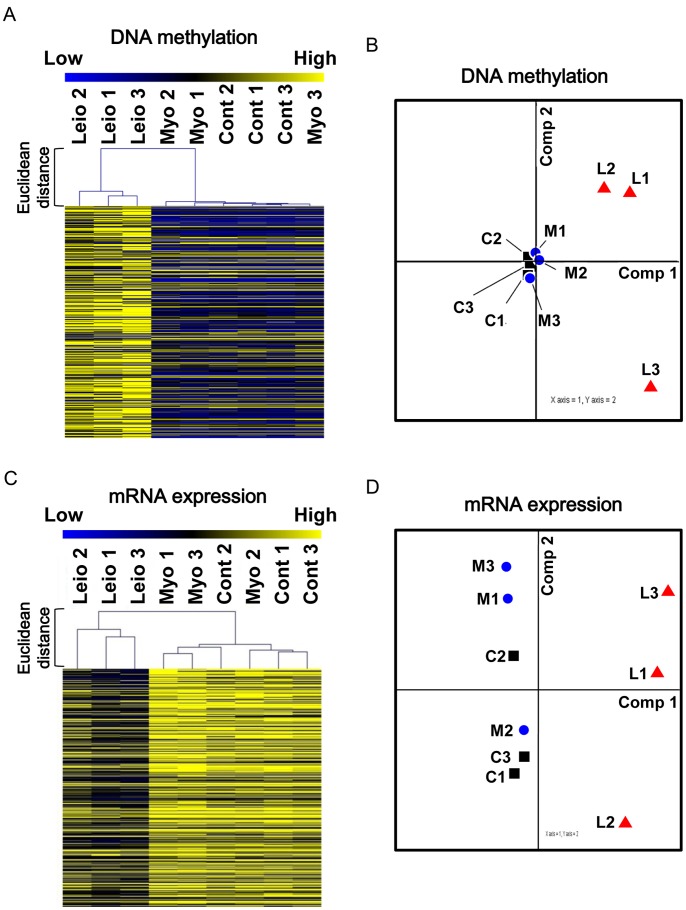
DNA methylation profiling and mRNA expression profiling of leiomyomas and myometrium with and without leiomyomas. DNA methylation profiles and mRNA expression profiles of leiomyomas (L1, L2 and L3), myometrium with leiomyomas (M1, M2 and M3) and myometrium without leiomyomas (C1, C2 and C3) were compared using hierarchical clustering analyses and principal component analyses. A: Hierarchical clustering analyses according to DNA methylation profiles. The Heat map in hierarchical clustering analysis indicates DNA methylation levels from unmethylated (blue) to completely methylated (yellow). Distances of DNA methylation pattern (Euclidean Distances) were calculated by MultiExperiment Viewer. B: Principal component analyses according to DNA methylation profiles. Vertical axis and horizontal axis show principal component numbers, respectively. The principal component analyses were performed using MultiExperiment Viewer. C: Hierarchical clustering analyses according to mRNA expression profiles. The Heat map in hierarchical clustering analysis indicates mRNA expression levels from low (blue) to high (yellow). Distances of mRNA expression pattern (Euclidean Distances) were shown on the left side. D: Principal component analyses according to mRNA expression profiles. Vertical axis and horizontal axis show principal component numbers, respectively.

To compare the genome-wide DNA methylation patterns among leiomyomas (L1, L2, and L3), the corresponding myometrium with leiomyomas (M1, M2, and M3), and the myometrium without leiomyomas (C1, C2, and C3), the hierarchical clustering was analyzed according to the DNA methylation status. Myometrium without leiomyomas (C1, C2, and C3) and myometrium with leiomyomas (M1, M2, and M3) showed a similar DNA methylation profiles, and these 6 tissues were classified into the same cluster group (Figure 1A). On the other hand, leiomyomas (L1, L2, and L3) were clustered separately from myometrium with or without leiomyomas (Figure 1A). The DNA methylation profiles of these 9 tissues were further compared with a principal component analysis. In Figure 1B, the component-1 and component-2 axes clearly distinguished leiomyomas (L1, L2, and L3) from myometrium with (M1, M2, and M3) or without (C1, C2, and C3) leiomyomas, while myometrium with leiomyomas (M1, M2, and M3) was very close to myometrium without leiomyomas (C1, C2, and C3). These results indicate that myometrium with and without leiomyomas have quite similar DNA methylation profiles whereas leiomyomas are clearly distinguished from the myometrium in terms of DNA methylation profiles.

We also analyzed the hierarchical clustering and principal components of the mRNA expression profiles. In the hierarchical clustering analysis, myometrium with and without leiomyomas were classified into the same cluster group, while leiomyomas were clearly separated from myometrium with or without leiomyomas (Figure 1C). In the principal component analysis, the component-1 axis clearly distinguished leiomyomas (L1, L2, and L3) from myometrium with (M1, M2, and M3) or without (C1, C2, and C3) leiomyomas, whereas myometrium with leiomyomas (M1, M2, and M3) and myometrium without leiomyomas (C1, C2, and C3) were not separated into the same group by the component-2 axis (Figure 1D). The data in Figures 1A and 1B suggest that profiling by DNA methylation is more useful than profiling by mRNA expression in defining cell identity.

### Analysis of DNA Methylation and mRNA Expression in Leiomyoma and Matched Adjacent Myometrium

**Figure 2:**
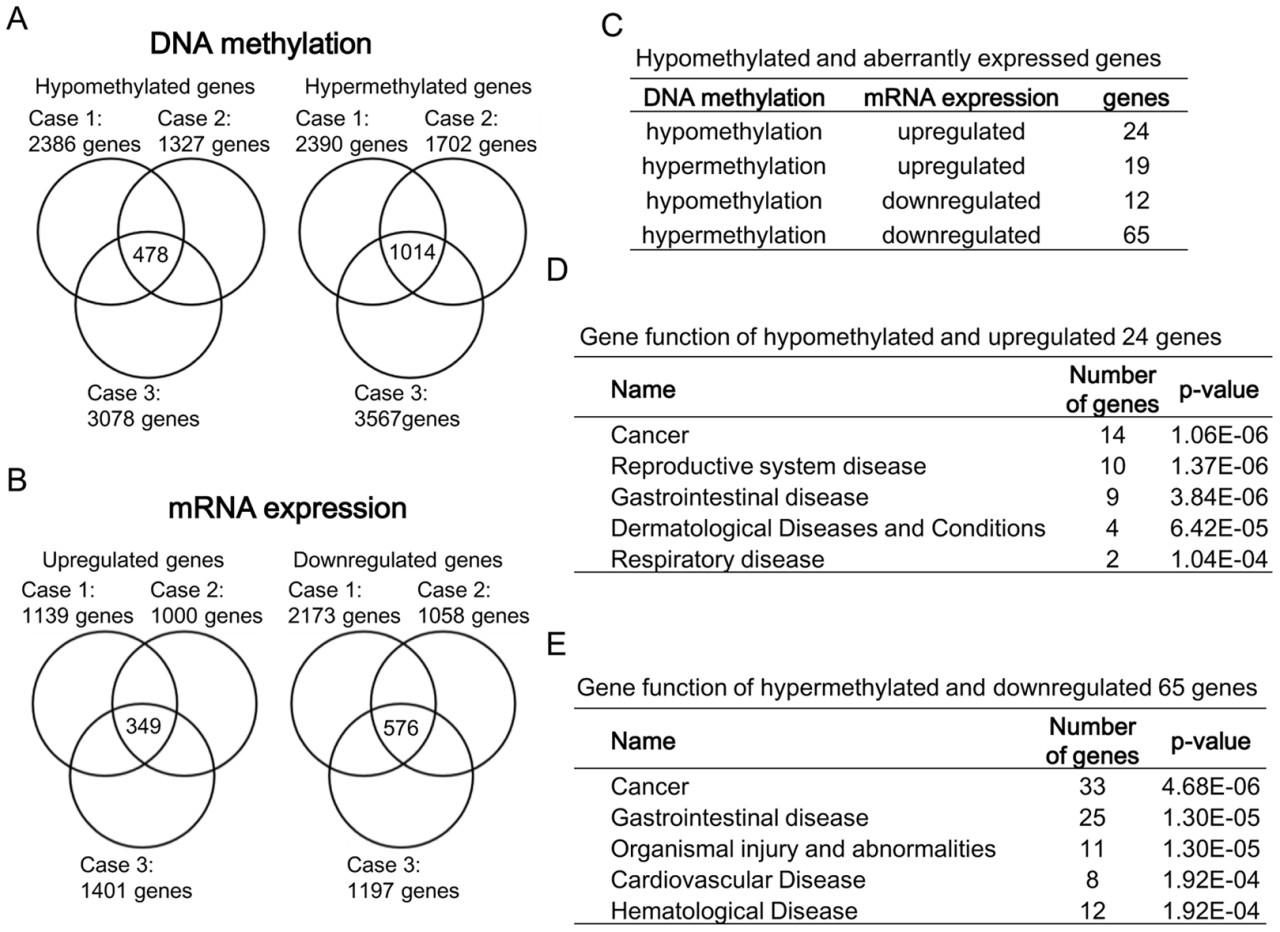
Aberrantly methylated or expressed genes in Leiomyomas. A: Venn diagrams show the number of aberrantly hypomethylated or hypermethylated genes in 3 paired samples. B: Venn diagrams show the number of aberrantly upregulated or downregulated genes in 3 paired samples. C: Integration of DNA methylation and mRNA expression data in uterine leiomyoma and adjacent myometrium. The numbers of genes which have both aberrant methylation and aberrant mRNA expression are shown. D: Functional analysis of 24 genes that have aberrant hypomethylation and upregulation of mRNA. The functional categories are shown with the number of included genes and p-values. Functional analysis was performed using IPA. E: Functional analysis of 65 genes that have aberrant hypermethylation and downregulation of mRNA. The functional categories are shown with the number of included genes and p-values. Functional analysis was performed using IPA.

We first compared the DNA methylation profiles between leiomyomas and adjacent myometrium in each of the three cases examined in this study. Case1, Case2 and Case3 had 2,386, 1,327 and 3,078 genes, respectively, that were less methylated in the leiomyomas than in the myometrium (Figure 2A, hypomethylated genes). Of these hypomethylated genes, 478 genes were shared by the three cases. Case1, Case2 and Case3 had 2,390, 1,702 and 3,567 genes, respectively, that were more methylated in the leiomyomas than in the myometrium (Figure 2A, hypermethylated genes). Of these hypermethylated genes, 1,014 genes were shared by the three cases.

Messenger RNA expression profiles were also analyzed among the three cases. Case1, Case2 and Case3 had 1,139, 1,000 and 1,401 genes, respectively, that were upregulated in the leiomyomas compared with the myometrium (Figure 2B, upregulated genes). Of these upregulated genes, 349 genes were shared by the three cases. Case1, Case2 and Case3 had 2,173, 1,058 and 1,197 genes, respectively, that were downregulated in the leiomyomas compared with the myometrium (Figure 2B, downregulated genes). Of these downregulated genes, 576 genes were shared by the three cases.

We then selected 120 genes that had both aberrant DNA methylation and aberrant mRNA expression. Of these genes, 24 genes were less methylated and transcriptionally upregulated in the leiomyomas compared with the myometrium, and 65 genes were more methylated and transcriptionally downregulated in the leiomyomas compared with the myometrium. According to the Ingenuity Pathways Analysis (IPA) [24], in the 24 hypomethylated and upregulated genes, the most specific pathway was “cancer process” in the section of diseases and disorders (Figure 2D). The genes included in the category “cancer process” are shown in Table 2. Collagen-related genes such as COL4A1 and COL4A2 have been previously reported to be upregulated in leiomyomas [25], [26], [27]. In the 65 hypermethylated and downregulated genes, the most specific pathway was also “cancer process” (Figure 2E). The genes included in the category “cancer process” are shown in Table 2. Glutathione S-transferase mu 5 (GSTM5) is a member of glutathione S-transferase family and protects cells as an anti-oxidant enzyme against reactive oxygen species. GSTM5 is also known to be involved in cancer development [28].

**Table 2:** Aberrantly methylated and expressed genes contained in "cancer process".

Gene symbol	Gene	fold change
**Hypomethylated and transcriptionally upregulated genes**		
CACNA1C	calcium channel, voltage-dependent, L type, alpha 1C subunit	1.974
COL4A1	collagen, type IV, alpha 1	1.822
COL4A2	collagen, type IV, alpha 2	2.022
COL6A3	collagen, type VI, alpha 3	1.950
CYP1B1	cytochrome P450, family 1, subfamily B, polypeptide 1	1.844
IRS1	insulin receptor substrate 1	2.395
KIAA1199	KIAA1199	8.507
POPDC2	popeye domain containing 2	4.952
PRL	prolactin	8.828
RAD51L1(RAD51B)	RAD51-like 1 (S. cerevisiae)	3.712
TACR2	tachykinin receptor 2	2.862
UNC5D	unc-5 homolog D (C. elegans)	3.851
VCAN	versican	2.677
WBSCR17	Williams-Beuren syndrome chromosome region 17	2.177
**Hypermethylated and transcriptionally downregulated genes**		
AIM1	absent in melanoma 1	−1.917
ANXA3	annexin A3	−4.190
CD74	CD74 molecule, major histocompatibility complex, class I	−1.620
CFB	complement factor B	−2.170
DAPK1	death-associated protein kinase 1	−2.007
DPP4	dipeptidyl-peptidase 4	−2.825
DUSP6	dual specificity phosphatase 6	−2.332
EFEMP1	EGF-containing fibulin-like extracellular matrix protein	−9.696
EPAS1	endothelial PAS domain protein 1	−2.369
EZR	ezrin	−2.227
FOXP1	forkhead box P1	−0.626
GATA2	GATA binding protein 2	−2.460
GSTM5	glutathione S-transferase mu 5	−9.998
HTATIP2	HIV-1 Tat interactive protein 2, 30 kDa	−3.253
IGF2BP3	insulin-like growth factor 2 mRNA binding protein 3	−1.684
KAT2B	K(lysine) acetyltransferase 2B	−2.715
KDR	kinase insert domain receptor	−2.565
LDB2	LIM domain binding 2	−2.034
LIMA1	LIM domain and actin binding 1	−1.759
MEOX2	mesenchyme homeobox 2	−1.850
MYEF2	myelin expression factor 2	−2.304
NR3C1	nuclear receptor subfamily 3, group C, member 1	−2.132
NR4A2	nuclear receptor subfamily 4, group A, member 2	−1.931
NR4A3	nuclear receptor subfamily 4, group A, member 3	−2.136
NR5A2	nuclear receptor subfamily 5, group A, member 2	−2.317
NRN1	neuritin 1	−3.641
NTRK2	neurotrophic tyrosine kinase, receptor, type 2	−2.175
NUAK1	NUAK family, SNF1-like kinase, 1	−2.568
PLCE1	phospholipase C, epsilon 1	−4.468
SDPR	serum deprivation response	−2.895
SORBS2	sorbin and SH3 domain containing 2	−2.291
SPTBN1	spectrin, beta, non-erythrocytic 1	−1.957
TIAM1	T-cell lymphoma invasion and metastasis 1	−1.730

The fold change was calculated as values of leiomyoma relative to adjacent myometrium in each case in the mRNA expression microarray.

### Validation of the Genes with Differential DNA Methylation and mRNA Expression

**Figure 3:**
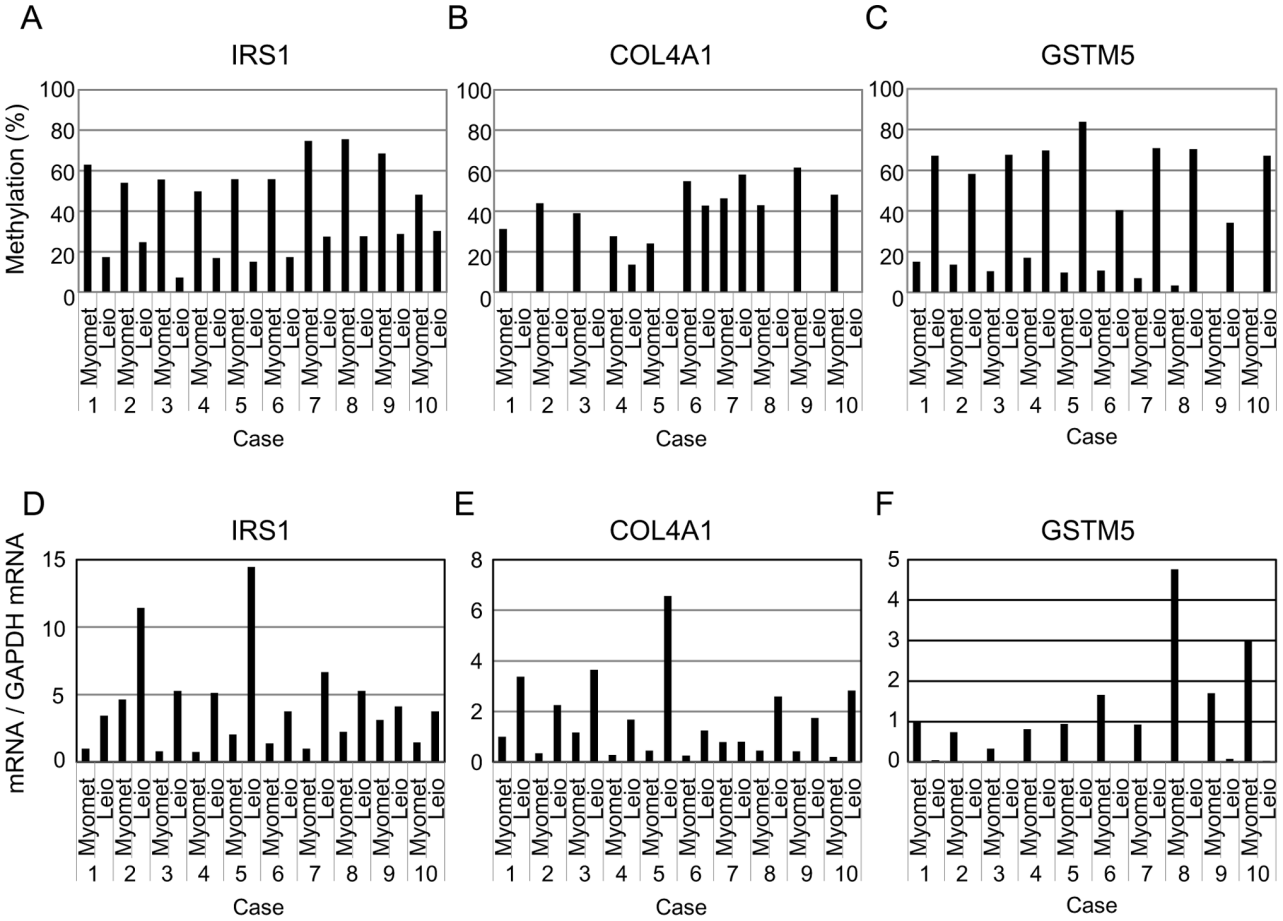
Analyses of DNA methylation levels and mRNA expression levels of IRS1, COL4A1 and GSTM5. DNA methylation levels and mRNA expression levels of IRS1, COL4A1 and GSTM5 were examined on 10 paired samples with myometrium (Myomet) and leiomyoma (Leio). DNA methylation levels of IRS1 (A), COL4A1 (B) and GSTM5 (C) were evaluated by bisulfite restriction mapping, quantified by densitometry, and expressed as %methylated DNA. The levels of mRNA expression of IRS1 (D), COL4A1 (E) and GSTM5 (F) were quantified by real-time quantitative RT-PCR and were normalized to GAPDH. The mRNA level in the myometrium of Case1 was expressed as 1.

Among the hypomethylated and upregulated genes, we focused on insulin receptor substrate 1 (IRS1) and COL4A1 which have aberrant DNA hypomethylation in the gene body closed to the promoter region. IRS1 is initially characterized as a cytosolic adaptor protein involved in insulin receptor (INSR) and insulin-like growth factor 1 receptor (IGF1R) signaling. More recently, it has been shown to be involved in proliferation and transformation of cancer cells [29], [30]. First, we examined the DNA methylation status of the IRS1 and COL4A1 using combined bisulfite restriction analysis in Case1, Case2, Case3 and additional 7 paired samples of leiomyoma and matched myometrium. As shown in Figure 3A, the methylation levels of the IRS1 were lower than those in myometrium in all the 10 samples. In addition, mRNA levels of IRS1 in leiomyomas were higher than those in myometrium in all the 10 samples (Figure 3D). In COL4A1, the methylation levels in leiomyomas were lower than those in myometrium in all samples except Case7 (Figure 3B), and mRNA levels in leiomyomas were higher than those in myometrium in all samples except Case7 (Figure 3E).

To examine the possibility that IRS1 mRNA expression is under the regulation of DNA methylation, immortalized human uterine smooth muscle cells (1×10^6^ cells/25 cm^2^ tissue culture flask) were treated by an inhibitor of DNA methylation (5-aza-dC, 1 uM) for 96 h. IRS1 mRNA levels were significantly (p<0.05) increased by 5-aza-dC, suggesting that DNA methylation is involved in the regulation of IRS1 mRNA expression (data not shown). The treatment with 5-aza-dC caused no significant changes in cell morphology and cell proliferation.

Among the hypermethylated and downregulated genes, we focused on GSTM5 which has aberrant DNA hypomethylation in the promoter region. As shown in Figure 3C and 3F, the methylation levels of the GSTM5 promoter in leiomyomas were higher than those in myometrium while mRNA levels of GSTM5 in leiomyomas were remarkably lower than those in myometrium in all the 10 samples.

### ER Alpha- target Genes with Aberrant DNA Methylation and mRNA Expression

In the aberrantly methylated and expressed 120 genes, 22 genes which have the consensus sequences of ER response element (ERE) in the promoter region (from −1,000 bp to the transcription start site) were extracted using the MAPPER database [20] (Table 3). In addition to COL4A1, COL6A3 and GSTM5, DAPK1 (death-associated protein kinase 1) and NUAK1 (novel kinase family 1), which were hypermethylated and transcriptionally downregulated, were included. DAPK1, which is a tumor suppressor gene, has been shown to be associated with apoptosis, and the downregulation and DNA hypermethylation were reported in several tumors [31], [32], [33], [34]. NUAK1 is known to possess tumor suppressive properties through the control of cellular senescence [35], [36].

**Table 3:** Estrogen receptor alpha-target genes with aberrant DNA methylation in the promoter and aberrant mRNA expression.

Gene		mRNA expression#
**Hypomethylated ERa target genes**		
COL4A1	collagen, type IV, alpha 1	↑
COL6A3	collagen, type VI, alpha 3	↑
RPL39	ribosomal protein L39	↑
ZMAT3	zinc finger, matrin-type 3	↑
OXTR	oxytocin receptor	↓
**Hypermethylated ERa target genes**		
BCAN	brevican	↑
KIF5C	kinesin family member 5C	↑
NPTX2	neuronal pentraxin II	↑
TFAP2C	transcription factor AP-2 gamma	↑
SCIN	scinderin	↑
THBS2	thrombospondin 2	↑
OCIAD2	OCIA domain containing 2	↓
CRHBP	corticotropin releasing hormone binding protein	↓
NUAK1	NUAK family, SNF1-like kinase, 1	↓
CCDC68	coiled-coil domain containing 68	↓
NR5A2	nuclear receptor subfamily 5, group A, member 2	↓
ELTD1	EGF, latrophilin and seven transmembrane domain containing 1	↓
FAM162B	family with sequence similarity 162, member B	↓
IGF2BP3	insulin-like growth factor 2 mRNA binding protein 3	↓
DAPK1	death-associated protein kinase 1	↓
GSTM5	glutathione S-transferase mu 5	↓
EZR	ezrin	↓

# Microarray mRNA expression statuses of leiomyoma relative to adjacent myometrium are shown.

### Analysis of X chromosome Genotype

**Figure 4:**
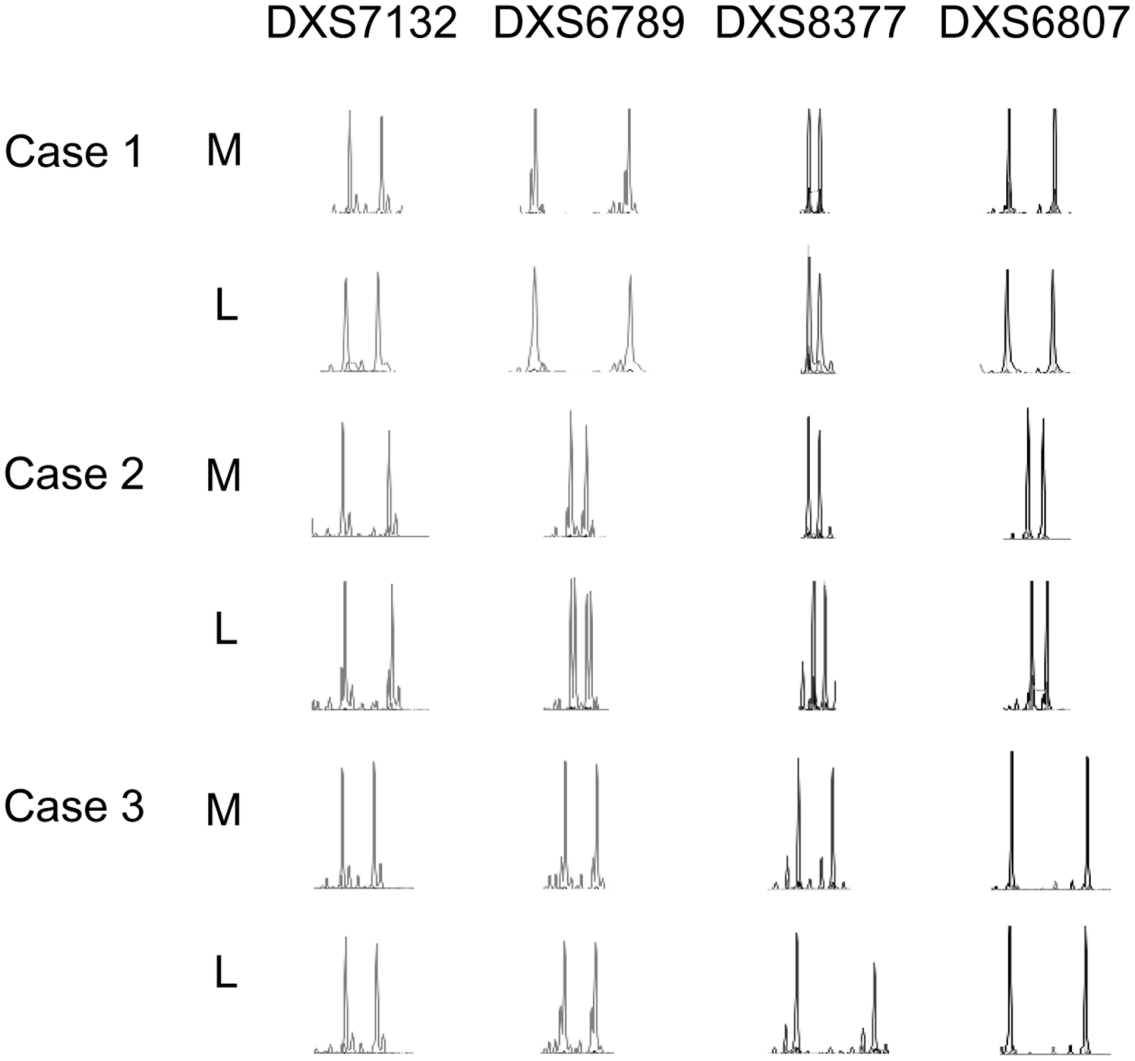
X chromosomal microsatellite analysis of leiomyoma and adjacent myometrium. Chromatographs using four tandem repeat markers (DXS7132, DXS6789, DXS8377 and DXS6807) are indicated. Chromatographs of DNA from leiomyoma (L) and adjacent myometrium (M) obtained from Case1 (Top), Case2 (Middle) and Case3 (Bottom) are shown. The PCR products of DXS7132, DXS6789, DXS8377 and DXS6807 were separated electrophoretically on a Fluorescent Capillary System ABI PRISM 310 and analyzed with GeneScan software (Applied Biosystems). The allelic status in each case was examined by comparing the allelic statuses of the leiomyoma and adjacent myometrium.

Recent reports have shown that in females, some cancer cells lose an inactive X chromosome and replicate an active X chromosome. It is called “loss-of-inactive X chromosome and gain-of-active X chromosome” [16], [17]. The cancer cells affected by this event have two active X chromosomes, resulting in the DNA hypomethylation status in X chromosome. Therefore, we decided to investigate whether uterine leiomyomas suffer this event and result in the hypomethylation status of X chromosome. Since the cells affected by this event should have two genetically same X chromosomes, we investigated the heterozygosity of X chromosomes in leiomyomas using following X-linked short tandem repeats markers: DXS7132, DXS6789, DXS8377 and DXS6807 spanning p and q arms. The result showed that all three cases showed heterozygosity in both myometrium and leiomyomas in all markers (Figure 4), indicating that both the leiomyoma and myometrium have a normal X chromosome genotype.

### Chromosome Distribution of Aberrantly Methylated CpG Sites

**Figure 5:**
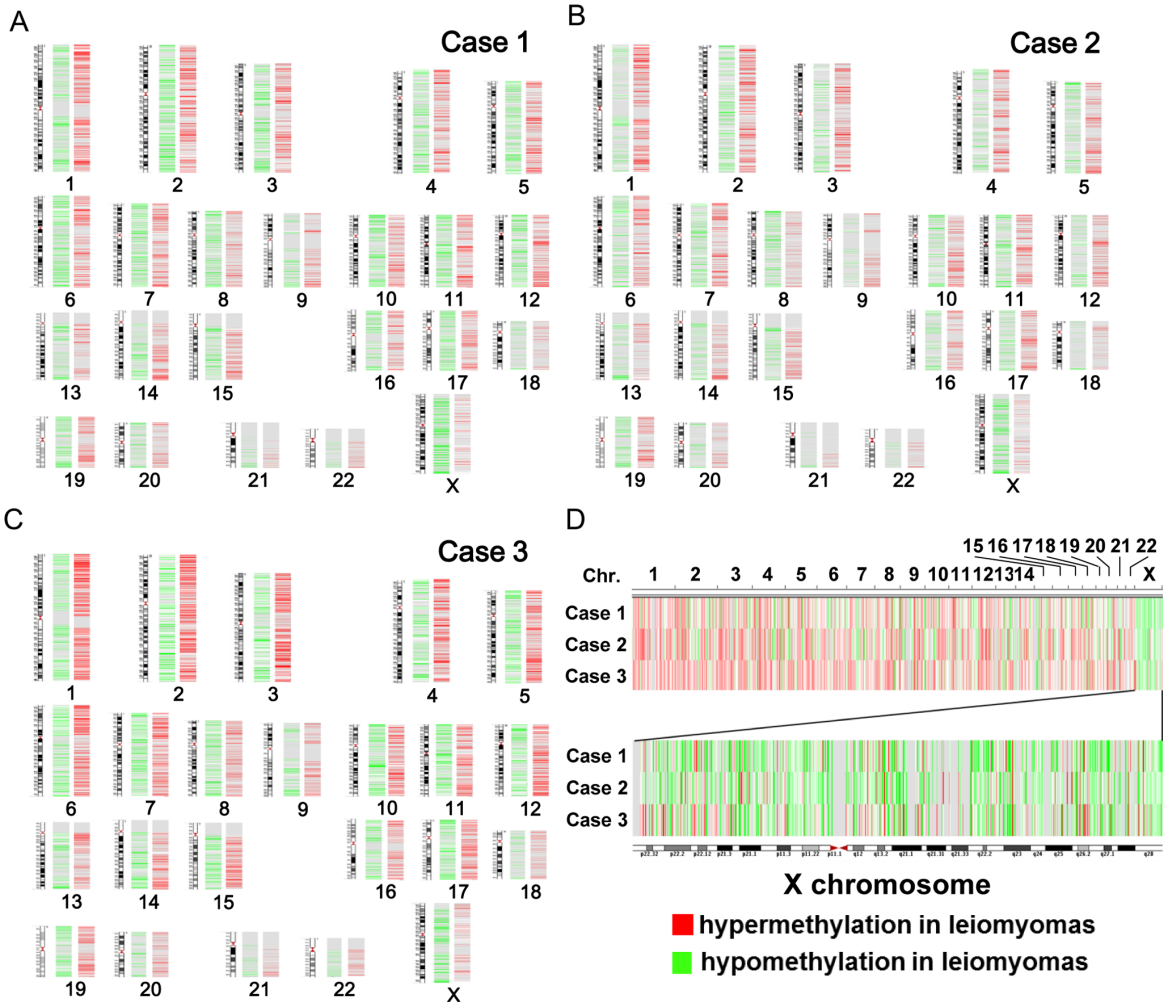
The chromosomal distribution of aberrantly methylated CpGs in uterine leiomyomas. The locations of CpGs, in which the difference of beta value between leiomyoma and adjacent myometrium are greater than 0.3, are shown with green bar (hypomethylated CpG in leiomyoma) or red bar (hypermethylated CpG in leiomyoma). The chromosomal distribution of aberrantly methylated CpGs was shown in Case1 (A), Case2 (B) and Case3 (C). Autosomal chromosomal number and sex chromosome are shown on the bottom. D: All chromosomes are aligned side by side in a row (Top), and X chromosome is shown in magnified scale (Bottom). Green bar (hypomethylated CpG in leiomyoma) and red bar (hypermethylated CpG in leiomyoma) are shown in the same line.

Aberrant hypomethylation and hypermethylation occurred on all chromosomes in all three cases (Figure 5). Hypermethylated CpG sites (red) were enriched compared with hypomethylated CpG sites (green) in the autosomes in all three cases (Figure 5). On the other hand, X chromosome was preferentially enriched with hypomethylated CpG sites (green) in all three cases with remarkably low p values (5.31E-95 in Case 1, 1.34E-55 in Case 2, 1.63E-14 in Case 3), and the distribution of the hypomethylated CpG sites was observed throughout the X chromosome (Figure 5).

## Discussion

We performed a genome-wide DNA methylation analysis in uterine leiomyomas using a method that interrogates over 450,000 CpG sites of DNA methylation statuses of consensus coding sequences. Our results suggest that uterine leiomyomas are associated with genome-wide alterations in DNA methylation at multiple gene promoter regions.

### Does the Adjacent Myometrium with Leiomyoma have the Potential to Develop into Uterine Leiomyomas?

Uterine leiomyomas often show multifocal tumorigenesis from the myometrium. We speculated that the adjacent myometrium, which looks normal, has the potential to develop into leiomyomas, i.e., already has aberrant DNA methylation. In this study, we selected paired tissue samples of leiomyoma and adjacent myometrium from women who had a single leiomyoma nodule. Interestingly, the DNA methylation status of the myometrium adjacent to the leiomyoma was quite similar to that in the normal uterine myometrium. This result suggests that adjacent myometrium with a leiomyoma nodule does not acquire the potential in DNA methylation levels to develop into leiomyomas. Another possibility is that cells with aberrant DNA methylation are present in the adjacent myometrium, but are too few to detect. In other words, aberrant DNA methylation may occur only in a limited number of cells. Recently, Ono et al. reported that side population cells in the myometrium and leiomyoma tissues act as tissue stem cells and have the potential to differentiate and proliferate [37], [38]. It has also been shown that each leiomyoma nodule shows monoclonal cell feature [39], indicating that each leiomyoma nodule is derived from just one affected cell as the origin of the tumor. Taken together, these findings suggest that tumor stem cells with aberrant DNA methylation are present in the myometrium and develop into leiomyoma nodules.

### Identification of Genes with Aberrant DNA Methylation that are Associated with Tumorigenesis of Uterine Leiomyomas

Of 120 genes that had both aberrant DNA methylation and aberrant mRNA expression in leiomyomas, 24 genes were less methylated and transcriptionally upregulated in the leiomyomas compared with the myometrium, and 65 genes were more methylated and transcriptionally downregulated in the leiomyomas compared with the myometrium. The biological relevance of these aberrantly methylated and concomitantly expressed genes was “cancer process” in the section of diseases and disorders according to IPA. The genes associated with “cancer process” contained potentially novel or relevant candidate genes for leiomyoma formation such as IRS1, COL4A1, COL4A2, COL6A3, and GSTM5.

Gene expression of IRS1 was found to be increased in uterine leiomyomas in this study. IRS1 has been shown to be involved in cancer progression and metastasis by mediating proliferative and anti-apoptotic functions of the INSR and IGF1R signaling [30], [40]. Recently, IRS1 has been found to have a novel function in addition to its role in intracellular signal transduction. Interestingly, nuclear presence of IRS1 contributes to malignant transformation [41]. Overexpression or ectopic expression of IRS1 causes cell transformation, including development of the ability to form colonies and tumors [42], [43]. In contrast, when IRS1 expression is experimentally decreased, cancer cells lose their transformed phenotype [44], [45], [46].

In the present study, the gene expressions of COL4A1, COL4A2 and COL6A3 were found to be increased in uterine leiomyomas. Extracellular matrix (ECM) plays an important role in the pathophysiology of uterine leiomyomas. Collagens are most predominant components of ECM of uterine leiomyomas [1]. Increased production of collagens contributes to the volume expansion and fibroid formation [1]. IRS1 is also reported to be involved in the upregulation of collagen genes, including COL4A1, COL4A2 and COL6A3, suggesting that IRS1 possibly contributes to the leiomyoma nodule formation by upregulating the gene expression of COL4A1, COL4A2, and COL6A3 [47].

On the other hand, gene expression of GSTM5 was decreased in uterine leiomyomas. GST family has been shown to play a crucial role in preventing DNA damage [48], [49], and overexpression of GST family inhibits oxidative stress-induced apoptosis and results in cell proliferation [48], [49]. Interestingly, GSTM5, one of mu-classes of GST family, may have a different function in tumorigenesis. Peng et al. [34] reported that promoter DNA hypermethylation and low mRNA expression of GSTM5 was observed in Barrett’s adenocarcinoma, and suggested that DNA hypermethylation of GSTM5 is an early event in carcinogenesis [28]. Aberrant DNA hypermethylation and downregulation of GSTM5 have also been reported in brain tumors, salivary gland cancers and leukemia [50], [51], [52]. However, the detailed role of GSTM5 in cancer development remains to be explored.

It is interesting to note that uterine leiomyomas develop only after menarche, indicating that growth of uterine leiomyomas depends on estrogen. We hypothesize that aberrant DNA methylation of the promoter regions of genes targeted by ER alpha causes an abnormal response to estrogen in uterine leiomyomas. Thus, tumor stem cells of leiomyomas in the myometrium may have become hyper-responsive or hypo-responsive to estrogen. In fact, exposure to environmental estrogen during reproductive tract development caused genes targeted by ER alpha to become hyper-responsive to estrogen in the adult myometrium [15]. The present study detected 22 ER alpha-target genes with both aberrant DNA methylation and aberrant mRNA expression, including COL4A1, COL6A3, DAPK1, and NUAK1. For example, if the promoter is aberrantly hypomethylated in ER alpha-target genes, ER alpha can bind to the promoter, resulting in a hyper-response to estrogen. In the case of the COL4A1 and COL6A3 genes, estrogen exposure causes overproduction of collagens after menarche. On the other hand, if the promoter is aberrantly hypermethylated in the ER alpha-target genes, ER alpha cannot bind to the promoter, resulting in a poor response to estrogen. In the case of DAPK1 and NUAK1, which are apoptosis-related genes that are induced by estrogen, apoptosis is not caused by estrogen, resulting in cell proliferation after menarche. Taken together, these findings suggest that aberrant responses of ER alpha-target genes to estrogen caused by aberrant DNA methylation in their promoter are involved in the development of uterine leiomyomas after menarche.

One may have a question whether the genes with aberrant expression in “cancer process” identified in uterine leiomyomas are also involved in the development of uterine leiomyosarcomas. Skubitz et al. previously reported aberrant overexpression of 16 genes in leiomyosarcomas [53], but those genes were different from the genes with aberrant expression in this study. Also, uterine leiomyosarcoma development is generally independent on estrogen exposure.

It is reported that tumor stem cells of uterine leiomyomas do not respond to estrogen because of low ER expression [38]. It is unclear how tumor stem cells of leiomyomas acquire the responsiveness to estrogen. Interestingly, recent evidence has shown that IRS1 regulates the transcription of ER alpha-target genes by forming a complex with ER alpha [54]. The IRS1/ER-alpha complex translocates into the nucleus and interacts with the promoter region containing ER-responsive elements, which in turn influences transcriptional activities via decreasing proteolytic turnover of ER alpha [54]. IRS1 also enhances ER alpha activities via the PI-3K/Akt pathway [55], [56]. From these findings, we speculate that tumor stem cells with overexpression of IRS1 respond to estrogen by acquiring ER alpha expression and stimulating ER alpha activities, resulting in the development of leiomyomas.

Recently, Navarro et al. reported 10 hypomethylated and transcriptionally upregulated genes, and 36 hypermethylated and transcriptionally downregulated genes in uterine leiomyomas compared with the corresponding myometrium using genome-wide approach [13]. However, the aberrant genes reported by them were not consistent with the genes identified in this study. There seem to be a number of differences between two studies. First, the method used for DNA methylation analysis differs. The genome-wide approach used by Navarro et al. covered a few CpG sites in the promoter region in each gene (1–2 CpG sites per gene) while our method can cover much more CpG sites (about 16 CpG sites per gene) broadly from the promoter to the gene body region. Second, the gene extraction algorithm is different between the two studies. We compared the difference in DNA methylation and mRNA expression in a paired sample in each case, and extracted the aberrantly methylated and differentially expressed gene, in which all the three cases showed common aberrations. On the other hand, Navarro et al. compared the difference in DNA methylation and mRNA expression between the uterine leiomyoma group and the myometrium group with the limma package which is based on t-statistic, but did not do in a paired sample in each case [13], [24]. Furthermore, we analyzed Japanese women who had a single leiomyoma nodule while Navarro et al. analyzed uterine leiomyomas with multiple nodules from African American women. More importantly, there must be inevitable differences in DNA methylation among individuals, which may be due to the difference in various local and environmental factors among individuals. In fact, we found that the DNA methylation status of the promoter region of ER alpha gene and GS20656 gene (differently methylated genomic loci in uterine leiomyomas) varies in even normal myometrium among individuals [11], [18], which may represent a within-physiological change in a certain cell type such as smooth muscle cells in myometrium [11], [18]. These differences between the two studies and the individual-related difference should be taken into consideration with some caution, and further studies are needed to make a more definitive conclusion.

### X Chromosome Genotyping and Chromosome Distribution of Aberrant DNA Methylation

In the present study, we used an advanced genome-wide DNA methylation analysis to confirm that hypomethylated-CpG sites were remarkably enriched on the X chromosome in uterine leiomyomas compared with adjacent myometrium. Loss of the inactive X chromosome and aberrant replication of the active X chromosome has been shown in several female-related cancer cells [16], [17]. Our analysis of the X chromosome genotype demonstrated that these events do not occur in uterine leiomyomas. The mechanism of DNA hypomethylation in the X chromosome in uterine leiomyomas is likely to be different from that in other cancer cells [16], [17]. We have suggested that X-inactivation machineries are disturbed in uterine leiomyomas [12]. However, the detailed mechanism of the enriched DNA hypomethylation in X chromosome in uterine leiomyomas, and the relationship between DNA hypomethylation status of X chromosome and the pathogenesis of uterine leiomyomas remain to be elucidated. In the course of this study, we identified 11 genes in the X chromosome of uterine leiomyomas that were hypomethylated in all three cases (data not shown). We are now examining these genes to see if they have roles in the pathogenesis of uterine leiomyomas.

### Conclusions

We propose the following hypothesis for pathogenesis of uterine leiomyomas: tissue stem cells in the myometrium transform to tumor stem cells by suffering genome-wide aberrant DNA methylation by unknown factors such as unfavorable environmental exposures. Aberrant DNA methylation and its related transcriptional aberration in cancer-related genes such as IRS1 may represent a critical initial mechanism that triggers transformation of a single tissue stem cell to a tumor stem cell, which will eventually develop into a monoclonal leiomyoma tumor. In addition, the aberrant DNA methylation of the promoter of ER alpha-target genes, e.g. COL4A1, COL6A3, DAPK1 and NUAK1, is responsible for the aberrant response to estrogen. The tumor stem cells aberrantly respond to estrogen after menarche, and gradually proliferate and differentiate to form leiomyoma nodules.

Our results suggest that aberrant DNA methylation contributes to the pathogenesis of uterine leiomyomas and may lead to the development of new strategies for treatment.

## References

[1] Stewart EA (2001) Uterine fibroids. Lancet 357: 293–298. 11214143 10.1016/S0140-6736(00)03622-9

[2] Bajekal N, Li TC (2000) Fibroids, infertility and pregnancy wastage. Hum Reprod Update 6: 614–620. 11129696 10.1093/humupd/6.6.614

[3] Chiaffarino F, Parazzini F, La Vecchia C, Chatenoud L, Di Cintio E, et al (1999) Diet and uterine myomas. Obstet Gynecol 94: 395–398. 10472866 10.1016/s0029-7844(99)00305-1

[4] Faerstein E, Szklo M, Rosenshein N (2001) Risk factors for uterine leiomyoma: a practice-based case-control study. I. African-American heritage, reproductive history, body size, and smoking. Am J Epidemiol 153: 1–10. 11159139 10.1093/aje/153.1.1

[5] Faerstein E, Szklo M, Rosenshein NB (2001) Risk factors for uterine leiomyoma: a practice-based case-control study. II. Atherogenic risk factors and potential sources of uterine irritation. Am J Epidemiol 153: 11–19. 11159140 10.1093/aje/153.1.11

[6] Shiota K, Yanagimachi R (2002) Epigenetics by DNA methylation for development of normal and cloned animals. Differentiation 69: 162–166. 11841471 10.1046/j.1432-0436.2002.690406.x

[7] Lieb JD, Beck S, Bulyk ML, Farnham P, Hattori N, et al (2006) Applying whole-genome studies of epigenetic regulation to study human disease. Cytogenet Genome Res 114: 1–15. 16717444 10.1159/000091922 PMC2734277

[8] Shiota K, Kogo Y, Ohgane J, Imamura T, Urano A, et al (2002) Epigenetic marks by DNA methylation specific to stem, germ and somatic cells in mice. Genes Cells 7: 961–969. 12296826 10.1046/j.1365-2443.2002.00574.x

[9] Iwatani M, Ikegami K, Kremenska Y, Hattori N, Tanaka S, et al (2006) Dimethyl sulfoxide has an impact on epigenetic profile in mouse embryoid body. Stem Cells 24: 2549–2556. 16840553 10.1634/stemcells.2005-0427

[10] Wu Q, Ohsako S, Ishimura R, Suzuki JS, Tohyama C (2004) Exposure of mouse preimplantation embryos to 2,3,7,8-tetrachlorodibenzo-p-dioxin (TCDD) alters the methylation status of imprinted genes H19 and Igf2. Biol Reprod 70: 1790–1797. 14960483 10.1095/biolreprod.103.025387

[11] Yamagata Y, Maekawa R, Asada H, Taketani T, Tamura I, et al (2009) Aberrant DNA methylation status in human uterine leiomyoma. Mol Hum Reprod 15: 259–267. 19218588 10.1093/molehr/gap010

[12] Maekawa R, Yagi S, Ohgane J, Yamagata Y, Asada H, et al (2011) Disease-dependent differently methylated regions (D-DMRs) of DNA are enriched on the X chromosome in uterine leiomyoma. J Reprod Dev 57: 604–612. 21685710 10.1262/jrd.11-035a

[13] Navarro A, Yin P, Monsivais D, Lin SM, Du P, et al (2012) Genome-wide DNA methylation indicates silencing of tumor suppressor genes in uterine leiomyoma. PLoS One 7: e33284. 22428009 10.1371/journal.pone.0033284 PMC3302826

[14] Hitchler MJ, Wikainapakul K, Yu L, Powers K, Attatippaholkun W, et al (2006) Epigenetic regulation of manganese superoxide dismutase expression in human breast cancer cells. Epigenetics 1: 163–171. 17965603 10.4161/epi.1.4.3401

[15] Greathouse KL, Bredfeldt T, Everitt JI, Lin K, Berry T, et al (2012) Environmental estrogens differentially engage the histone methyltransferase EZH2 to increase risk of uterine tumorigenesis. Mol Cancer Res 10: 546–557. 22504913 10.1158/1541-7786.MCR-11-0605 PMC3879949

[16] Kawakami T, Zhang C, Taniguchi T, Kim CJ, Okada Y, et al (2004) Characterization of loss-of-inactive X in Klinefelter syndrome and female-derived cancer cells. Oncogene 23: 6163–6169. 15195139 10.1038/sj.onc.1207808

[17] Sirchia SM, Ramoscelli L, Grati FR, Barbera F, Coradini D, et al (2005) Loss of the inactive X chromosome and replication of the active X in BRCA1-defective and wild-type breast cancer cells. Cancer Res 65: 2139–2146. 15781624 10.1158/0008-5472.CAN-04-3465

[18] Asada H, Yamagata Y, Taketani T, Matsuoka A, Tamura H, et al (2008) Potential link between estrogen receptor-alpha gene hypomethylation and uterine fibroid formation. Mol Hum Reprod 14: 539–545. 18701604 10.1093/molehr/gan045

[19] Saeed AI, Sharov V, White J, Li J, Liang W, et al (2003) TM4: a free, open-source system for microarray data management and analysis. Biotechniques 34: 374–378. 12613259 10.2144/03342mt01

[20] Marinescu V, Kohane I, Riva A (2005) The MAPPER database: a multi-genome catalog of putative transcription factor binding sites. Nucleic Acids Res 33: D91–97. 15608292 10.1093/nar/gki103 PMC540057

[21] Huang da W, Sherman BT, Tan Q, Kir J, Liu D, et al (2007) DAVID Bioinformatics Resources: expanded annotation database and novel algorithms to better extract biology from large gene lists. Nucleic Acids Res 35: W169–175. 17576678 10.1093/nar/gkm415 PMC1933169

[22] Robinson JT, Thorvaldsdottir H, Winckler W, Guttman M, Lander ES, et al (2011) Integrative genomics viewer. Nat Biotechnol 29: 24–26. 21221095 10.1038/nbt.1754 PMC3346182

[23] Kumaki Y, Oda M, Okano M (2008) QUMA: quantification tool for methylation analysis. Nucleic Acids Res 36: W170–175. 18487274 10.1093/nar/gkn294 PMC2447804

[24] Feng G, Du P, Krett NL, Tessel M, Rosen S, et al (2010) A collection of bioconductor methods to visualize gene-list annotations. BMC Res Notes 3: 10. 20180973 10.1186/1756-0500-3-10 PMC2829581

[25] Vanharanta S, Wortham NC, Laiho P, Sjoberg J, Aittomaki K, et al (2005) 7q deletion mapping and expression profiling in uterine fibroids. Oncogene 24: 6545–6554. 15940248 10.1038/sj.onc.1208784

[26] Gilden M, Malik M, Britten J, Delgado T, Levy G, et al.. (2012) Leiomyoma fibrosis inhibited by liarozole, a retinoic acid metabolic blocking agent. Fertil Steril. 10.1016/j.fertnstert.2012.07.1132 22925684

[27] Weston G, Trajstman AC, Gargett CE, Manuelpillai U, Vollenhoven BJ, et al (2003) Fibroids display an anti-angiogenic gene expression profile when compared with adjacent myometrium. Mol Hum Reprod 9: 541–549. 12900513 10.1093/molehr/gag066

[28] Peng DF, Razvi M, Chen H, Washington K, Roessner A, et al (2009) DNA hypermethylation regulates the expression of members of the Mu-class glutathione S-transferases and glutathione peroxidases in Barrett’s adenocarcinoma. Gut 58: 5–15. 18664505 10.1136/gut.2007.146290 PMC2845391

[29] Morelli C, Garofalo C, Sisci D, del Rincon S, Cascio S, et al (2004) Nuclear insulin receptor substrate 1 interacts with estrogen receptor alpha at ERE promoters. Oncogene 23: 7517–7526. 15318176 10.1038/sj.onc.1208014

[30] Esposito DL, Aru F, Lattanzio R, Morgano A, Abbondanza M, et al (2012) The insulin receptor substrate 1 (IRS1) in intestinal epithelial differentiation and in colorectal cancer. PLoS One 7: e36190. 22558377 10.1371/journal.pone.0036190 PMC3338610

[31] Martoriati A, Doumont G, Alcalay M, Bellefroid E, Pelicci PG, et al (2005) dapk1, encoding an activator of a p19ARF-p53-mediated apoptotic checkpoint, is a transcription target of p53. Oncogene 24: 1461–1466. 15608685 10.1038/sj.onc.1208256

[32] Raval A, Tanner SM, Byrd JC, Angerman EB, Perko JD, et al (2007) Downregulation of death-associated protein kinase 1 (DAPK1) in chronic lymphocytic leukemia. Cell 129: 879–890. 17540169 10.1016/j.cell.2007.03.043 PMC4647864

[33] Claus R, Hackanson B, Poetsch AR, Zucknick M, Sonnet M, et al (2012) Quantitative analyses of DAPK1 methylation in AML and MDS. Int J Cancer 131: E138–142. 21918973 10.1002/ijc.26429 PMC3463871

[34] Missaoui N, Hmissa S, Trabelsi A, Traore C, Mokni M, et al (2011) Promoter hypermethylation of CDH13, DAPK1 and TWIST1 genes in precancerous and cancerous lesions of the uterine cervix. Pathol Res Pract 207: 37–42. 21129853 10.1016/j.prp.2010.11.001

[35] Bernard D, Augert A (2010) NUAK1 links genomic instability and senescence. Aging (Albany NY) 2: 317–319. 20603521 10.18632/aging.100153 PMC2919246

[36] Hou X, Liu JE, Liu W, Liu CY, Liu ZY, et al (2011) A new role of NUAK1: directly phosphorylating p53 and regulating cell proliferation. Oncogene 30: 2933–2942. 21317932 10.1038/onc.2011.19

[37] Ono M, Maruyama T, Masuda H, Kajitani T, Nagashima T, et al (2007) Side population in human uterine myometrium displays phenotypic and functional characteristics of myometrial stem cells. Proc Natl Acad Sci U S A 104: 18700–18705. 18003928 10.1073/pnas.0704472104 PMC2141840

[38] Ono M, Qiang W, Serna VA, Yin P, Coon JSt, et al (2012) Role of stem cells in human uterine leiomyoma growth. PLoS One 7: e36935. 22570742 10.1371/journal.pone.0036935 PMC3343011

[39] Zhang P, Zhang C, Hao J, Sung CJ, Quddus MR, et al (2006) Use of X-chromosome inactivation pattern to determine the clonal origins of uterine leiomyoma and leiomyosarcoma. Hum Pathol 37: 1350–1356. 16949924 10.1016/j.humpath.2006.05.005

[40] White MF (2002) IRS proteins and the common path to diabetes. Am J Physiol Endocrinol Metab 283: E413–422. 12169433 10.1152/ajpendo.00514.2001

[41] Reiss K, Del Valle L, Lassak A, Trojanek J (2012) Nuclear IRS-1 and cancer. J Cell Physiol 227: 2992–3000. 22454254 10.1002/jcp.24019 PMC3615708

[42] DeAngelis T, Chen J, Wu A, Prisco M, Baserga R (2006) Transformation by the simian virus 40 T antigen is regulated by IGF-I receptor and IRS-1 signaling. Oncogene 25: 32–42. 16170362 10.1038/sj.onc.1209013

[43] Valentinis B, Romano G, Peruzzi F, Morrione A, Prisco M, et al (1999) Growth and differentiation signals by the insulin-like growth factor 1 receptor in hemopoietic cells are mediated through different pathways. J Biol Chem 274: 12423–12430. 10212216 10.1074/jbc.274.18.12423

[44] Cesarone G, Garofalo C, Abrams MT, Igoucheva O, Alexeev V, et al (2006) RNAi-mediated silencing of insulin receptor substrate 1 (IRS-1) enhances tamoxifen-induced cell death in MCF-7 breast cancer cells. J Cell Biochem 98: 440–450. 16440325 10.1002/jcb.20817

[45] del Rincon SV, Guo Q, Morelli C, Shiu HY, Surmacz E, et al (2004) Retinoic acid mediates degradation of IRS-1 by the ubiquitin-proteasome pathway, via a PKC-dependant mechanism. Oncogene 23: 9269–9279. 15516986 10.1038/sj.onc.1208104

[46] D’Ambrosio C, Keller SR, Morrione A, Lienhard GE, Baserga R, et al (1995) Transforming potential of the insulin receptor substrate 1. Cell Growth Differ 6: 557–562. 7647039

[47] Tseng YH, Butte AJ, Kokkotou E, Yechoor VK, Taniguchi CM, et al (2005) Prediction of preadipocyte differentiation by gene expression reveals role of insulin receptor substrates and necdin. Nat Cell Biol 7: 601–611. 15895078 10.1038/ncb1259

[48] Strange RC, Spiteri MA, Ramachandran S, Fryer AA (2001) Glutathione-S-transferase family of enzymes. Mutat Res 482: 21–26. 11535245 10.1016/s0027-5107(01)00206-8

[49] Townsend DM, Tew KD (2003) The role of glutathione-S-transferase in anti-cancer drug resistance. Oncogene 22: 7369–7375. 14576844 10.1038/sj.onc.1206940 PMC6361125

[50] Etcheverry A, Aubry M, de Tayrac M, Vauleon E, Boniface R, et al (2010) DNA methylation in glioblastoma: impact on gene expression and clinical outcome. BMC Genomics 11: 701. 21156036 10.1186/1471-2164-11-701 PMC3018478

[51] Bell A, Bell D, Weber RS, El-Naggar AK (2011) CpG island methylation profiling in human salivary gland adenoid cystic carcinoma. Cancer 117: 2898–2909. 21692051 10.1002/cncr.25818 PMC3123690

[52] Kearns PR, Chrzanowska-Lightowlers ZM, Pieters R, Veerman A, Hall AG (2003) Mu class glutathione S-transferase mRNA isoform expression in acute lymphoblastic leukaemia. Br J Haematol 120: 80–88. 12492580 10.1046/j.1365-2141.2003.04039.x

[53] Skubitz K, Skubitz A (2003) Differential gene expression in leiomyosarcoma. Cancer 98: 1029–1038. 12942572 10.1002/cncr.11586

[54] Morelli C, Garofalo C, Bartucci M, Surmacz E (2003) Estrogen receptor-alpha regulates the degradation of insulin receptor substrates 1 and 2 in breast cancer cells. Oncogene 22: 4007–4016. 12821935 10.1038/sj.onc.1206436

[55] Sun M, Paciga JE, Feldman RI, Yuan Z, Coppola D, et al (2001) Phosphatidylinositol-3-OH Kinase (PI3K)/AKT2, activated in breast cancer, regulates and is induced by estrogen receptor alpha (ERalpha) via interaction between ERalpha and PI3K. Cancer Res 61: 5985–5991. 11507039

[56] Campbell RA, Bhat-Nakshatri P, Patel NM, Constantinidou D, Ali S, et al (2001) Phosphatidylinositol 3-kinase/AKT-mediated activation of estrogen receptor alpha: a new model for anti-estrogen resistance. J Biol Chem 276: 9817–9824. 11139588 10.1074/jbc.M010840200

